# Prevalence and Association of Transfusion Transmitted Infections with ABO and Rh Blood Groups among Blood Donors in the Western Region of Saudi Arabia: A 7-Year Retrospective Analysis

**DOI:** 10.3390/medicina58070857

**Published:** 2022-06-27

**Authors:** Malik A. Altayar, Mohammed M. Jalal, Ahmed Kabrah, Fadi S. I. Qashqari, Naif A. Jalal, Hani Faidah, Mohammed A. Baghdadi, Saeed Kabrah

**Affiliations:** 1 Department of Medical Laboratory Technology, Faculty of Applied Medical Sciences, University of Tabuk, Tabuk 71491, Saudi Arabia; maltayar@ut.edu.sa (M.A.A.); mjalal@ut.edu.sa (M.M.J.); 2 Laboratory Medicine Department, Faculty of Applied Medical Sciences, Umm Al-Qura University, Makkah 21955, Saudi Arabia; amkabrah@uqu.edu.sa; 3 Department of Microbiology, Faculty of Medicine, Umm Al-Qura University, Makkah 21955, Saudi Arabia; fsqashqari@uqu.edu.sa (F.S.I.Q.); najalal@uqu.edu.sa (N.A.J.); hsfaidah@uqu.edu.sa (H.F.); 4 King Faisal Specialist Hospital and Research Centre (KFSH & RC), Jeddah 23431, Saudi Arabia; mbaghdadi@kfshrc.edu.sa

**Keywords:** ABO blood groups, blood donors, KFSH and RC, Saudi Arabia, blood transfusion-transmitted infections, TTIs

## Abstract

This study was aimed at determining the prevalence estimate and association of transfusion-transmitted infections (TTIs) with ABO and Rh blood groups among blood donors at the King Faisal Specialist Hospital and Research Center (KFSH & RC) in the western region of Saudi Arabia. A retrospective study was conducted at the blood bank center of KFSH and RC from 1 January 2013 to 31 December 2019. Data on ABO and Rh blood group testing, serological testing, molecular investigations, serological assays, nucleic acid testing (NATs), and socio-demographic information were gathered. During the study period, there were 959,431 blood donors at the KFSH and RC. The overall 7-year cumulative prevalence estimate of blood transfusion-transmitted infections among blood donors was low at 7.93%, with an average prevalence estimate of 0.66%. Donors with the O blood group, the O RhD +ve blood group, in particular, were more at risk of developing TTIs, whereas donors with the AB blood group, the AB RhD −ve blood group, in particular, were at the lowest risk of developing TTIs. In total, 96.9% of the blood donors were males (*n* = 916,567). Almost half of the blood donors belong to the O blood group (49.4%). A total of 861,279 (91.0%) donors were found to be RhD positive. The percentages of TTIs were found to be higher in RhD +ve donors compared with RhD −ve donors. The prevalence estimate of the hemoglobin C (HbC) infection was the most common TTI among the blood donors being 3.97%, followed by malaria being 2.21%. The least prevalence estimate of TTI in the present study was for NAT HIV being 0.02%. Significant associations were observed between RhD +ve and RhD −ve among the malaria-infected donors (A: χ^2^ = 26.618, *p* = 0.001; AB: χ^2^ = 23.540, *p* = 0.001; B: χ^2^ = 5.419, *p* = 0.020; O: χ^2^ = 68.701, *p* = 0.001). The current 7-year retrospective study showed a low level of TTIs among blood donors. However, we urge that more research encompassing the entire country be conducted in order to obtain more representative results in terms of the prevalence estimate and association of transfusion-transmitted infections with ABO and Rh blood groups in communities.

## 1. Introduction

The polymorphic features of people are represented by blood group antigens. Variations in blood group antigen expression may increase or decrease the host’s susceptibility to a variety of infections [1]. The fact that blood groups can operate as receptors/coreceptors for bacteria, viruses, and parasites illustrates the importance of blood groups in infection.

Additionally, the antigens of some blood groups allow for cell adsorption, signal transduction, and/or the retention of membrane micro-domains. Blood types can also affect an infection’s innate immune response [2,3].

The most common blood group system is the ABO Blood Group System. The inheritance of A and B alleles is responsible for the distribution of ABO blood types among individuals [4].

ABO glycosyltransferase can then use H antigen as a substrate. The addition of 1–2 fucose by FUT1 or H-glycosyltransferase results in the formation of H antigen. Individuals in Group A express 1-3 N-acetylgalactosamine (GalNAc), while those in Group B express 1–3 galactose (Gal). Individuals of Group O, on the other hand, have dormant ABO genes and only express the H-antigen precursor [2]. Naturally occurring ABO system antibodies against the antigens of the ABO blood group have been stimulated by microbes and A/B-antigen-like environmental material [5]. ABO antibodies are part of the body’s innate immune system and fight harmful bacteria and viruses that carry ABO-active antigens. Blood groups, on the other hand, have the ability to act as false receptors. Bacteria, viruses, and parasites employ certain blood groups as receptors and ligands. For example, the Duffy blood group antigen is a receptor for different malarial parasites, such as Plasmodium vivax [6,7].

According to research, ABO antigens can also prevent TTI agents from binding to the polysaccharide. Cells that lack these antigens, on the other hand, are in danger of contracting transfusion-transmissible infections (TTIs) [2,4]. Hepatitis C virus (HCV), the hepatitis B virus (HBV), and the human immunodeficiency virus (HIV) are the leading causes of death in the world [8]. Globally, it was reported that 36.70 million people are infected with HIV infection, 25.70 million people are infected with HBV infection, and 71.0 million people are infected with HCV. It was assessed that 2.30 million and 2.70 million people are infected with HIV/HCV and HIV/HBV co-infection due to the same mode of transmission [9]. In Tuscany, Italy, males gained 2.9 years, and females gained 2.6 years in life expectancy at birth. Infectious disease mortality increased by 0.11 years for males and 0.16 years for females, resulting in a loss of 0.11 years of life expectancy for males and 0.16 years for females [10]. According to a study conducted in Pakistan, the prevalence rate of hepatitis B and hepatitis C was 6.7% and 14.3%, respectively, with 80% co-morbidities of HIV/HCV and 20% of HBV/HCV [11,12]. Individuals with a chronic hepatitis B infection have a higher risk of developing liver cirrhosis and cancer, as well as a variety of other disorders. This infection is extremely contagious [13]. Due to their similar viral transmission paths, HCV and HBV infections are well-known among HIV patients. In HIV-infected patients, co-morbidities such as liver issues caused by HCV or HBV infection are a major concern [14]. Syphilis is caused by the bacteria *Treponema pallidum*, which can infect a person by penetrating the oral lining of the vaginal area. *Syphilis* has become more common worldwide, particularly among homosexual men and HIV donors [15].

The association of various blood groups with some human pathogens causes health-related hazards, particularly TTIs such as hepatitis B virus (HBV), hepatitis C virus (HCV), human immunodeficiency virus (HIV) and human T-lymphotropic virus (HTLV), as blood transfusion is one of the modes of transmission of these infectious agents [16,17]. Because of their link to specific disorders, blood group distribution plays a significant role in blood transfusion [18,19]. For example, the O blood group has been linked to an increased frequency of HBV, although there was no significant link between the four types of ABO blood groups in the case of HCV [20].

To the best of our knowledge, there is a lack of studies on the prevalence and association of transfusion-transmitted infections with ABO and Rh Blood groups among blood donors in the western region of Saudi Arabia. Therefore, the current retrospective study is the first attempt to identify the prevalence and association of transfusion-transmitted infections with ABO and Rh blood groups among blood donors at the KFSH and RC, which may contribute to improving and optimizing blood transfusion services not only in the studied region but also in other Saudi Arabian regions.

## 2. Materials and Methods

### 2.1. Study Design and Period

A 7-year retrospective study was conducted from 1 January 2013 to 31 December 2019.

### 2.2. Study Population and Eligibility Criteria

All eligible blood donors’ data that fulfilled the national blood bank selection criteria were included in this study. These criteria were based on a predefined measure: (1) age between 18 and 65 years old, (2) weight > 50 kg, and (3) no medical history. Donors’ information was obtained from the donor registry. The information collected from the database included year, sex, age, blood groups, type of donation, frequency of blood donation, and the outcome variables of TTIs markers.

### 2.3. Laboratory Tests

According to the manufacturer’s recommendations, all donors were tested for ABO, Rh and TTIs markers using ELISA and molecular NAT kits.

Micro-agglutination tests (BioRad, Hercules, CA, USA) were used to evaluate donor blood groups using forward (cell grouping using anti-A and anti-B antisera) and reverse grouping (serum grouping using A and B red cells) methods. The final blood group is confirmed only if both forward and reverse groups are identical. The Rh group was also determined by micro-agglutination tests utilizing anti-D reagents, and the Coombs test was performed to detect weak D antigen.

Following the manufacturer’s instructions, immunoassay analyses ArchitectPlus kits (Abbott Laboratories, Chicago, IL, USA) were used to screen serum individually for the presence of hepatitis B surface antigen (HBsAg), antibody to hepatitis B surface antigen (HBsAb), hepatitis B core antibody (HBcAb), HCV antibody (anti-HCV), human immunodeficiency virus 1&2 antigen/antibody combo (HIV1/2 Ag/Ab), antihuman T-cell lymphotropic virus 1 and 2 (anti-HTLV-1&2), and syphilis (SYPH). Antibody for malaria was tested using an automated immunochemistry analyzer (EtiMax 3000, DiaSorin, Saluggia, Italy).

The donors’ samples were tested in triplicate to rule out false-positive or false-negative results. Additionally, all donor samples were tested in a pool of four using nucleic acid testing (NAT) for HCV, HBV and HIV (Cobas6800, Roche, Basel, Switzerland).

Positive serology or molecular results were repeated with a new sample and tested individually. The prevalence estimates of various blood types and TTIs were determined and compared. TTIs were also examined in relation to the ABO and Rh blood categories among blood donors.

### 2.4. Ethical Consideration

The study protocol was approved by the Institutional Review Board of KFSH and RC, Jeddah, Saudi Arabia (RC-J/45/40).

### 2.5. Data Analysis

Data retrieved from the KFSH and RC Blood Bank and Pathological Laboratory Data Management Systems were transferred to Excel spreadsheets (Microsoft Corp., Redmond, WA, USA). For data analysis, the Statistical Package for Social Science (SPSS version 28) for Mac (New York, NY, USA) was used. The two-tailed paired *t*-test and linear regression analysis were used to determine statistical significance. A *p*-value of 0.05 was considered statistically significant.

## 3. Results

### 3.1. Characteristics of the Included Blood Donors

There were 959,431 blood donors at the KFSH and RC during the study period, with the highest number of donors documented in 2016 (149,866) and the least number of donors documented in 2013 (114,741). The average number of donors per year was determined to be 135,169. Due to missing or incomplete data, 13,246 (1.4%) were excluded, whereas 946,185 (98.6%) were included in the current study. In total, 68.1% of blood donors were Saudi (*n* = 644,142) and 31.9% were from other nationalities (*n* = 302,043). In total, 96.9% of blood donors were males (*n* = 916,567) and only 3.1% were females (*n* = 29,618). Almost half of the blood donors belong to the O blood group (49.4%), while the AB blood group showed the smallest percentage of donors (4.7%). Out of the 946,185 donors, 861,279 were found to be RhD positive. The majority of blood donors (93.3%) experienced repeated blood donations (Table 1).

### 3.2. Distribution of Transfusion-Transmitted Infections Marker among Donors Based on ABO and Rh Blood Groups

The overall 7-year cumulative prevalence estimate of TTIs among blood donors was low at 7.93%, with an average prevalence estimate of 0.66%. The study showed that donors with the O blood group were more at risk of developing TTIs as 37,522 (3.9%) donors were reported as positive TTIs, followed by blood group A (2.3%), blood group B (1.4%), and blood group AB (0.34%). In terms of the RhD antigen, the O RhD +ve blood group was more at risk of developing hepatitis TTIs as 34,248 (3.6%) donors were reported as positive TTIs, followed by A RhD +ve blood group (2.1%), B RhD +ve blood group (1.2%), O RhD −ve blood group (0.34%), AB RhD +ve blood group (0.31%), A RhD −ve blood group (0.16%), B RhD −ve blood group (0.09%), and AB RhD −ve blood group (0.03%).

The prevalence estimate of the HBcAb was the most common TTIs marker among the blood donors being 3.97%, followed by malaria being 2.21%. The least prevalence estimate of TTI in the present study was for NAT HIV being 0.02%. The percentages of TTIs infection were found to be higher in RhD +ve donors compared with RhD −ve donors. The highest percentage of HBcAb infection (1.8859%) was found among the donors who had the O RhD +ve blood group, while the lowest (0.0122%) was among donors who had the AB RhD −ve blood group. Similarly, the highest percentage of malaria infection (1.2423%) was found among the donors who had the O RhD +ve blood group, while the lowest (0.0093%) was among donors who had the AB RhD −ve blood group (Table 2).

### 3.3. The Association between RhD Positive and RhD Negative among the Positive Transfusion-Transmitted Infected Donors

Significant associations were observed between RhD +ve and RhD −ve among the malaria infected donors (A: χ^2^ = 26.618, *p* = 0.001; AB: χ^2^ = 23.540, *p* = 0.001; B: χ^2^ = 5.419, *p* = 0.020; O: χ^2^ = 68.701, *p* = 0.001). Significant associations were found between RhD +ve and RhD −ve among the NAT HCV infected donors (A: χ^2^ = 40.512, *p* = 0.001, AB: χ^2^ = 16.878, *p* = 0.001, O: χ^2^ = 5.823, *p* = 0.006). Similarly, significant associations were found between RhD +ve and RhD −ve among the NAT HBV infected donors (A: χ^2^ = 9.773, *p* = 0.002, AB: χ^2^ = 41.484, *p* = 0.001, O: χ^2^ = 15.155, *p* = 0.001). Significant associations were found between RhD +ve and RhD −ve among the SYPH infected donors (A: χ^2^ = 7.625, *p* = 0.006, O: χ^2^ = 63.942, *p* = 0.001). Significant associations were found between RhD +ve and RhD −ve among the HTLV infected donors (A: χ^2^ = 19.050, *p* = 0.001, AB: χ^2^ = 4.225, *p* = 0.040, O: χ^2^ = 21.224, *p* = 0.001, respectively). Significant associations were found between RhD +ve and RhD −ve among the HBsAg infected donors (A: χ^2^ = 3.941, *p* = 0.047, AB: χ^2^ = 32.739, *p* = 0.001, O: χ^2^ = 7.928, *p* = 0.005). A significant association was found between RhD +ve and RhD −ve among the HIV-infected donors (B: χ^2^ = 5.189, *p* = 0.023). Significant associations were found between the RhD +ve and RhD −ve among the HCV infected donors (A: χ^2^ = 6.95, *p* = 0.014, and AB: χ^2^ = 4.726, *p* = 0.030, respectively). Significant associations were found between RhD +ve and RhD −ve among the HBcAb reactive donors (AB: χ^2^ = 6.425, *p* = 0.011, B: χ^2^ = 33.595, *p* = 0.001, χ^2^ = 76.805, O: *p* = 0.000). Significant associations were found between the RhD +ve and RhD −ve among the HBsAb reactive donors (A: χ^2^ = 4.470, *p* = 0.034, and B: χ^2^ = 11.469, *p* = 0.001).

However, no significant association was observed between RhD +ve and RhD –ve among the NAT HIV-infected donors (ABO: *p* > 0.05). No significant association was observed between RhD +ve and RhD −ve among the HIV and HBcAb reactive donors (A: *p* > 0.05). No significant association was observed between RhD +ve and RhD −ve among the SYPH, HIV, and HBsAb reactive donors (AB: *p* > 0.05). No significant associations were found between RhD +ve and RhD −ve among the NAT HIV, NAT HCV, NAT HBV, SYPH, HTLV, HBsAg, and anti-HCV reactive donors (B: *p* > 0.05). No significant associations were observed between RhD +ve and RhD −ve among the HIV, HCV, and HBsAb reactive donors (O: *p* > 0.05) (Table 3).

## 4. Discussion

To our knowledge, the current study is the first comprehensive investigation to evaluate the prevalence and association of transfusion-transmitted infections with ABO and Rh blood groups among blood donors in the western region of Saudi Arabia. The allocation of blood into ABO and Rh groups is commonly conducted in the KFSH and RC blood bank center, as well as other blood banks worldwide, to match compatible blood required in blood transfusion, organ transplant, and to prevent hemolytic diseases among newborns, as well as in numerous legal matters linked with forensic medicine and medico-legal cases, including resolving paternity conflicts [16].

The predominant blood group in the present study was O, and the least common was AB, which correlates with the finding of a recent systematic review by Saeed Kabrah and his colleagues showed that the distribution pattern of the ABO blood group among the Saudi population was O > A > B > AB [21]. Several studies conducted in different African and European countries were in line with our findings, as the O blood group was the most prevalent and the AB blood group was the least prevalent [19,20]. Similarly, blood group O was the most common in the study, including all newly accepted health students in a large university in the Eastern Province of Saudi Arabia, and AB was the least common [18].

The demographic pattern of our study sample showed that 96.9% of blood donors were males, and only 3.1% were females. Likewise, the study conducted in the Central Region of Saudi Arabia reported that 82.98% of the donors were male [22]. Almost identical findings have also been reported in studies conducted in Brazil (99.6%) [23], Ethiopia (86.8%) [17], Cameroon (82.0%) [24], Nigeria (81.9%) [25], and Coastal South India (95.2%) [26]. This could be attributed to the cultural stigma in some regions that women should not donate blood because they already lose blood on a monthly basis due to menstruation, and donating more blood may cause weakness and endanger their health. In contrast, other studies from different contexts showed that the frequency of both male and female blood donors was nearly equal. For instance, the United States (51.7%), Belgium (54.6%), the United Kingdom (47.0%), the Netherlands (50.0%), Spain (54.0%), France (50.0%), Finland (45.0%), and Denmark (50.0%) [27,28,29,30,31,32].

Our study showed that 91.0% of blood donors were Rhesus positive. The results of other previous studies conducted in Saudi Arabia were consistent with our findings, as Rhesus positivity in blood donors was dominant [18,22].

Although the overall 7-year cumulative prevalence estimate of TTIs among blood donors was low at 7.93%, with an average prevalence estimate of 0.66%, an adequate understanding of TTIs in blood donors is still essential to further reducing patient mortality and morbidity. Our finding was higher than the result of the study conducted in the Central Region of Saudi Arabia, which revealed that the overall cumulative frequency of TTIs in the blood of donors was 1.002% [22]. In comparison to other Asian and African studies, such as Equatorial New Guinea (18.7%) [33], Mozambique (37.39%) [34] and Burkina Faso (24%) [35], our findings demonstrated a lower prevalence estimate of TTIs. The lower prevalence estimate of TTIs in the current investigation could be due to several factors, including the relatively low prevalence of blood-borne pathogens such as HIV among the population of Saudi Arabia, strict adherence to and implementation of guidelines designed to prevent the transmission of blood-borne pathogens, pre-marital screening, and pre-donation screening intended to screen out high-risk donors. Furthermore, the prevalence estimate of TTIs in our study was lower than in Sub-Saharan African countries, which could be attributed to these African region’s poor socioeconomic conditions, which make it difficult to provide adequate health care and invest in developing standardized blood transfusion infrastructure in accordance with WHO guidelines [36].

The association and susceptibility of blood group systems, especially ABO, to various disorders have been investigated and reported. According to the findings of a recent study conducted among blood donors at the National Blood Bank in Amman, Jordan, to determine the prevalence and association of transfusion-transmitted infections with ABO and Rh blood groups, O and A were the most common blood groups (37.44% and 36.82%), respectively, followed by B (18.62%) and AB (7.12%). When comparing the prevalence estimate of Rh +ve and Rh −ve blood donors, it was discovered that Rh +ve donors were more common (88.73%) than Rh −ve donors (11.27%). The most common viral marker was HBsAg (0.38%), followed by anti-HCV (0.13%), syphilis (0.02%), and anti-HIV (0.006%), and male donors were more infected than female donors [37]. One recent example was the association of ABO groups with COVID-19, suggesting that those with group A and AB are more prone to the infection, while those with group O are less susceptible [38]. This study showed that donors with the O blood group, particularly those with O RhD +ve blood group, were more at risk of developing TTIs, whereas donors with the AB blood group, the AB RhD −ve blood group, in particular, were at the lowest risk of developing TTIs. According to a study by Farwa Sijjeel et al., donors with blood group O were found to be highly contaminated with TTIs [13]. On the other hand, donors with blood group A were shown to be at a higher risk of developing HBV and HIV, while those with group O showed no significant association with TTIs in research conducted in Peshawar, Pakistan [39].

Our study showed that the HBcAb was the most common TTI marker among the blood donors, followed by malaria. However, an earlier study carried out in Saudi Arabia found that HBV is the most prevalent type of TTIs [21]. Similarly, a retrospective study conducted in Eritrea revealed the same result, with HBV being the highest [40]. Despite significant progress in Saudi Arabia to reduce HBV prevalence during the last 30 years, the number of HBV diagnosed cases have been consistent over the previous ten years, and it remains a persistent concern [41]. Because blood is a limited and expensive human resource, it should be used wisely and correctly. Prescription decisions should be based on national guidelines for the clinical use of blood, taking into account the individual patient’s needs with the least amount of cost and waste and the highest level of safety and efficacy [42]. Continuous improvement and implementation of donor selection, sensitive screening tests, and effective inactivation methods can ensure that the risk of developing TTIs is eliminated, or at the very least, reduced [43]. Moreover, community-based TTI awareness campaigns, emphasizing the diseases’ transmission and chronic nature, as well as emphasizing the significance of TTI vaccination in the general population, can all help to reduce the prevalence of TTIs in the Saudi community.

There are some limitations to the current study that will need to be addressed in future research. The retrospective aspect of this study limits its epidemiological importance. Furthermore, it renders the inclusions of all risk factors associated with TTIs. As a result, more research is needed to investigate the clinical association between antigen receptors in the O and A blood groups and infection and the pathogenesis and its association with the blood group. Obtaining data for 946,185 donors is a good consequence, but processing these data is critical. On the one hand, it is the total number of donors’ results, not the number of samples. In the current study, confirmatory tests were performed on donors with reactive serological and molecular markers, as the Saudi Ministry of Health recommended. Under these conditions, the calculations of prevalence, percentage, and the related statistical tests represent the estimated prevalence. On the other hand, the current study evaluates the association between the antigens of the ABO and RhD system. Further study can be conducted to estimate the difference between these blood groups and TTIs.

ABO antigens and Rh antigens are produced by genetically different entities whose properties, chemical nature, etc., are fundamentally different even if present on the same blood cells. Among other things, the genes responsible for the synthesis of ABH antigens, on the one hand, and RhD, on the other hand, are located on different chromosomes. This means that it is not fair to consider the “ABORhD” phenotype as a single and homogeneous entity, but, on the contrary, it is more sensible to analyze the results, on the one hand, according to the four groups of blood, A, B, AB, and O, and on the other hand, depending on the presence or absence of the RhD antigen. This mode of analysis also has the advantage of being more readable and more efficient from a statistical point of view.

## 5. Conclusions

The current 7-year retrospective study showed a low level of TTIs among blood donors at the King Faisal Specialist Hospital and Research Centre (KFSH and RC) in the western region of Saudi Arabia, which could be due to the good blood banking practice and guidelines of the hospital along with overall public awareness toward TTIs. However, there is still a need for improvement in terms of blood bank infrastructure and adherence to international blood bank criteria. The frequency rate of the O blood group was the highest among the blood donors, while the AB blood group was the lowest. The majority of blood donors showed positive Rh. The frequency rates of TTIs were found to be higher in RhD +ve donors compared with RhD −ve donors. HBcAb marker was the most common TTIs marker among the blood donors, followed by malaria infection. Significant associations were observed between RhD +ve and RhD –ve among the malaria-infected donors. Significant associations were found between RhD +ve and RhD −ve among the NAT HCV and NAT HBV infected donors. Furthermore, the percentage of female blood donors was much lower than that of male donors, which should be addressed and improved by public education for women about the necessity and benefits of blood donations in order to urge them to donate blood. Such knowledge is essential for the timely delivery of transfusion services to vulnerable patients and the coordination of blood blank inventories. We urge that more research encompassing the entire country be conducted in order to obtain more representative results in terms of ABO and Rh blood type system frequency rates, as well as the prevalence rate of TTIs in communities. This would be extremely helpful in developing a database for Saudi Arabia’s blood banks.

## Figures and Tables

**Table 1:** Study sample characteristics (*n* = 946,185).

Variable	Response Option	*n*	%
Year	2013	114,741	12.1
	2014	117,172	12.4
	2015	145,286	15.4
	2016	149,886	15.8
	2017	145,573	15.4
	2018	147,133	15.6
	2019	126,394	13.4
	Total	946,185	100.1
Nationality	Saudi	644,142	68.1
	Non-Saudi	302,043	31.9
	Total	946,185	100
Gender	Female	29,618	3.1
	Male	916,567	96.9
	Total	946,185	100
ABO	A	277,026	29.3
	AB	44,018	4.7
	B	157,837	16.7
	O	467,304	49.4
	Total	946,185	100
RhD	Negative	84,906	9.0
	Positive	861,279	91.0
	Total	946,185	100
Type of Donor	Repeated donor	882,608	93.3
	New donor	63,577	6.7
	Total	946,185	100

**Table 2:** Distribution of transfusion-transmitted infections markers among donors based on ABO and Rh blood groups (*n* = 946,185).

TTIs Markers			ABO																Prevalence		
			A				AB				B				O						
			RhD −ve		RhD +ve		RhD −ve		RhD +ve		RhD −ve		RhD +ve		RhD −ve		RhD +ve		RhD −ve	RhD +ve	Total
			*n*	(%)	*n*	(%)	*n*	(%)	*n*	(%)	*n*	(%)	*n*	(%)	*n*	(%)	*n*	(%)			
**Serological**	**Anti-Malaria**	**−ve**	21,060 ^a^	2.2258	250,185 ^b^	26.4414	3158 ^a^	0.3338	40,128 ^b^	4.2410	12,999 ^a^	1.3738	141,502 ^b^	14.9550	46,009 ^a^	4.8626	408,535 ^b^	43.1771	2.2119	0.1776	2.2119
		**+ve**	343 ^a^	0.0363	5438 ^b^	0.5747	88 ^a^	0.0093	644 ^b^	0.0681	243 ^a^	0.0257	3093 ^b^	0.3269	1006 ^a^	0.1063	11,754 ^b^	1.2423			
	**SYPH**	**−ve**	21351 ^a^	2.2565	255,208 ^b^	26.9723	3246 ^1^	3.4307	40,730 ^a^	4.3047	13,218 ^a^	1.3970	144,408 ^a^	15.2621	46,873 ^a^	4.9539	419,676 ^b^	44.3545	0.1328	0.0231	0.1328
		**+ve**	52 ^a^	0.0055	415 ^b^	0.0439	0 ^1^	0.0001	42 ^a^	0.0044	24 ^a^	0.0025	187 ^a^	0.0198	142 ^a^	0.0150	613 ^b^	0.0648			
	**Anti-HTLV-1/-2**	**−ve**	21363 ^a^	2.2578	255,393 ^b^	26.9919	3246 ^1^	3.4307	40,719 ^a^	4.3035	13,230 ^a^	1.3982	144,457 ^a^	15.2673	46,959 ^a^	4.9630	420,032 ^b^	44.3922	0.0717	0.0115	0.0717
		**+ve**	40 ^a^	0.0042	230 ^b^	0.0243	0 ^1^	0.0001	53 ^a^	0.0056	12 ^a^	0.0013	138 ^a^	0.0146	56 ^a^	0.0059	257 ^b^	0.0272			
	**HIV Ag/Ab**	**−ve**	21,388 ^a^	2.2604	255,459 ^a^	26.9988	3246 ^1^	3.4307	40,745 ^a^	4.3062	13,230 ^a^	1.3982	144,530 ^b^	15.2750	46,999 ^a^	4.9672	420,069 ^a^	44.3961	0.0503	0.0047	0.0503
		**+ve**	15 ^a^	0.0016	164 ^a^	0.0173	0^1^	0.0001	27 ^a^	0.0029	12 ^a^	0.0013	65 ^b^	0.0069	16 ^a^	0.0017	220 ^a^	0.0233			
	**Anti-HCV**	**−ve**	21,326 ^a^	2.2539	254,396 ^b^	26.8865	3221 ^a^	0.3404	40,573 ^b^	4.2881	13,186 ^a^	1.3936	144,112 ^a^	15.2308	46,859 ^a^	4.9524	418,736 ^a^	44.2552	0.3659	0.0332	0.3659
		**+ve**	77 ^a^	0.0081	1227 ^b^	0.1297	25 ^a^	0.0026	199 ^b^	0.0210	56 ^a^	0.0059	483 ^a^	0.0510	156 ^a^	0.0165	1553 ^a^	0.1641			
	**HBsAg**	**−ve**	21,372 ^a^	2.2588	255,090 ^b^	26.9598	3230 ^a^	0.3414	40,728 ^b^	4.3044	13,227 ^a^	1.3979	144,370 ^a^	15.2581	46,941 ^a^	4.9611	419,823 ^b^	44.3701	0.1340	0.0144	0.1340
		**+ve**	31 ^a^	0.0033	533 ^b^	0.0563	16 ^a^	0.0017	44 ^b^	0.0047	15 ^a^	0.0016	225 ^a^	0.0238	74 ^a^	0.0078	466 ^b^	0.0493			
	**HBcAb**	**−ve**	20,468 ^a^	2.1632	244,803 ^a^	25.8726	3131 ^a^	0.3309	38,940 ^b^	4.1155	12,740 ^a^	1.3465	137,483 ^b^	14.5302	45,419 ^a^	4.8002	402,445 ^b^	42.5334	3.9747	0.3327	3.9747
		**+ve**	935 ^a^	0.0988	10,820 ^a^	1.1435	115 ^a^	0.0122	1832 ^b^	0.1936	502 ^a^	0.0531	7112 ^b^	0.7517	1596 ^a^	0.1687	17,844 ^b^	1.8859			
	**HBsAb**	**−ve**	21,388 ^a^	2.2604	255,312 ^b^	26.9833	3246 ^1^	3.4307	40,769 ^a^	4.3088	13,241 ^a^	1.3994	144,448 ^b^	15.2664	46,944 ^a^	4.9614	419,695 ^a^	44.3565	0.1115	0.0093	0.1115
		**+ve**	15 ^a^	0.0016	311 ^b^	0.0329	0 ^1^	0.0001	3 ^a^	0.0003	1 ^a^	0.0001	147 ^b^	0.0155	71 ^a^	0.0075	594 ^a^	0.0628			
**Molecular (NAT)**	**HIV**	**−ve**	21,402 ^a^	2.2619	255,570 ^a^	27.0106	3246 ^1^	3.4307	40,772 ^1^	43.0910	13,242 ^1^	13.9953	144,568 ^a^	15.2790	47,001 ^a^	4.9674	420,203 ^a^	44.4102	0.0176	0.0018	0.0176
		**+ve**	1 ^a^	0.0001	53 ^a^	0.0056	0 ^1^	0.0001	0 ^1^	0.0001	0 ^1^	0.0001	27 ^a^	0.0029	14 ^a^	0.0015	86 ^a^	0.0091			
	**HCV**	**−ve**	21,403 ^1^	22.6204	255,140 ^a^	26.9651	3232 ^a^	0.3416	40,717 ^b^	4.3033	13,230 ^a^	1.3982	144,455 ^a^	15.2671	46,943 ^a^	4.9613	419,814 ^b^	44.3691	0.1219	0.0105	0.1219
		**+ve**	0 ^1^	0.0001	483 ^a^	0.0510	14 ^a^	0.0015	55 ^b^	0.0058	12 ^a^	0.0013	140 ^a^	0.0148	72 ^a^	0.0076	475 ^b^	0.0502			
	**HBV**	**−ve**	21,385 ^a^	2.2601	255,175 ^b^	26.9688	3231 ^a^	0.3415	40,740 ^b^	4.3057	13,227 ^a^	1.3979	144,447 ^a^	15.2663	46,944 ^a^	4.9614	419,903 ^b^	44.3785	0.1072	0.0126	0.1072
		**+ve**	18 ^a^	0.0019	448 ^b^	0.0473	15 ^a^	0.0016	32 ^b^	0.0034	15 ^a^	0.0016	148^a^	0.0156	71 ^a^	0.0075	386 ^b^	0.0408			
	Overall 7-year cumulative prevalence estimate of TTIs among blood donors																		7.2995	0.6313	7.9308

Note: Values in the same row and sub-table not sharing the same subscript are significantly different at *p* < 0.05 in the two-sided test of equality for column proportions. Cells with no subscript are not included in the test. Tests assume equal variances. −ve = negative and +ve = positive. ^a^ This category is not used in comparisons because its column proportion is equal to zero or one. ^b^ Tests are adjusted for all pairwise comparisons within a row of each innermost sub-table using the Benjamini–Hochberg correction.

**Table 3:** Results of the association between RhD positive and RhD negative within positive transfusion-transmitted infections markers (*n* = 946,185).

TTIs		Statistical Tests	ABO			
			A	AB	B	O
			RhD	RhD	RhD	RhD
**Serological**	**Anti-Malaria**	χ^2^	26.618	23.540	5.419	68.701
		*p*-Value	0.001 *	0.001 *	0.020 *	0.001 *
	**SYPH**	χ^2^	7.625	3.347	2.449	63.942
		*p*-Value	0.006 *	0.067 ^a^	0.118	0.001 *
	**anti-HTLV-1&2**	χ^2^	19.050	4.225	0.030	21.224
		*p*-Value	0.001 *	0.040 *^,a^	0.863	0.001 *
	**HIV1/2 Ag/A**b	χ^2^	0.107	2.151	5.189	2.809
		*p*-Value	0.743	0.142 ^a^	0.023 *	0.094
	**anti-HCV**	χ^2^	6.095	4.726	2.815	1.649
		*p*-Value	0.014 *	0.030 *	0.093	0.199
	**HBsAg**	χ^2^	3.941	32.739	1.432	7.928
		*p*-Value	0.047 *	0.001 *^,a^	0.231	0.005 *
	**HBcAb**	χ^2^	0.896	6.425	33.595	76.805
		*p*-Value	0.344	0.011 *	0.001 *	0.000 *
	**HBsAb**	χ^2^	4.470	0.239	11.469	0.279
		*p*-Value	0.034 *	0.625 ^a,b^	0.001 *	0.597
**Molecular (NAT)**	**HIV**	χ^2^	2.614		2.473	1.715
		*p*-Value	0.106 ^a^		0.116 ^a^	0.190
	**HCV**	χ^2^	40.512	16.878	0.048	5.823
		*p*-Value	0.001 *	0.001 *	0.826	0.016 *
	**HBV**	χ^2^	9.773	41.484	0.140	15.155
		*p*-Value	0.002 *	0.001 *^,a^	0.708	0.001 *

Results are based on nonempty rows and columns in each innermost sub-table. RhD denotes RhD +ve and RhD −ve. * The Chi-square statistic (χ^2^) is significant at the *p*-value = 0.05 level. ^a^ More than 20% of cells in this subtable have expected cell counts of less than 5. ^b^ The minimum expected cell count in this sub-table is less than one.

## Data Availability

The data that support the findings of this study are available from the corresponding author upon reasonable request.
